# Excitatory and inhibitory neuron defects in a mouse model of *Scn1b*‐linked EIEE52

**DOI:** 10.1002/acn3.51205

**Published:** 2020-09-26

**Authors:** Jacob M. Hull, Heather A. O’Malley, Chunling Chen, Yukun Yuan, Nicholas Denomme, Alexandra A. Bouza, Charles Anumonwo, Luis F. Lopez‐Santiago, Lori L. Isom

**Affiliations:** ^1^ Neuroscience Graduate Program University of Michigan Medical School Ann Arbor MI 48109 USA; ^2^ Department of Pharmacology University of Michigan Medical School Ann Arbor MI 48109 USA

## Abstract

**Objective:**

Human variants in voltage‐gated sodium channel (VGSC) α and β subunit genes are linked to developmental and epileptic encephalopathies (DEEs). Inherited, biallelic, loss‐of‐function variants in *SCN1B*, encoding the β1/β1B subunits, are linked to early infantile DEE (EIEE52). *De novo*, monoallelic variants in *SCN1A* (Nav1.1), *SCN2A* (Nav1.2), *SCN3A* (Nav1.3), and *SCN8A* (Nav1.6) are also linked to DEEs. While these VGSC‐linked DEEs have similar presentations, they have diverse mechanisms of altered neuronal excitability. Mouse models have suggested that *Scn2a*‐, *Scn3a*‐, and *Scn8a*‐linked DEE variants are, in general, gain of function, resulting in increased persistent or resurgent sodium current (I_Na_) and pyramidal neuron hyperexcitability. In contrast, *Scn1a*‐linked DEE variants, in general, are loss‐of‐function, resulting in decreased I_Na_ and hypoexcitability of fast‐spiking interneurons. VGSC β1 subunits associate with Nav1.1, Nav1.2, Nav1.3, and Nav1.6 and are expressed throughout the brain, raising the possibility that insults to both pyramidal and interneuron excitability may drive EIEE52 pathophysiology.

**Methods:**

We investigated excitability defects in pyramidal and parvalbumin‐positive (PV +) interneurons in the *Scn1b* ^−/−^ model of EIEE52. We also used *Scn1b^FL/FL^* mice to delete *Scn1b* in specific neuronal populations.

**Results:**

*Scn1b* ^−/−^ cortical PV + interneurons were hypoexcitable, with reduced I_Na_ density. *Scn1b* ^−/−^ cortical pyramidal neurons had population‐specific changes in excitability and impaired I_Na_ density. *Scn1b* deletion in PV + neurons resulted in 100% lethality, whereas deletion in Emx1 + or Camk2a + neurons did not affect survival.

**Interpretation:**

This work suggests that *SCN1B*‐linked DEE variants impact both excitatory and inhibitory neurons, leading to the increased severity of EIEE52 relative to other DEEs.

## Introduction

Voltage‐gated sodium channels (VGSCs) are responsible for action potential (AP) initiation and propagation in the nervous system.^1^ VGSCs in brain are heterotrimeric protein complexes composed of one pore‐forming α subunit and two nonpore‐forming β subunits.^2^, ^3^ Variants in VGSC α and β subunit genes are linked to the developmental and epileptic encephalopathies (DEEs). The DEE Dravet syndrome (DS) is linked to *de novo*, monoallelic, loss‐of‐function (LOF) variants in *SCN1A*, encoding Na_v_1.1, although rare gain‐of‐function (GOF) variants have been reported.^4^ *De novo*, monoallelic variants in *SCN2A* (Nav1.2), *SCN3A* (Nav1.3), and *SCN8A* (Nav1.6) are linked to other DEEs, including Ohtahara syndrome and early infantile epileptic encephalopathy type 13 (EIEE13).^5^, ^6^, ^7^, ^8^

Evidence from transgenic mouse models of DS supports the hypothesis that haploinsufficiency of *Scn1a* drives cortical and hippocampal GABAergic interneuron hypoexcitability, leading to disinhibition.^9^, ^10^, ^11^ Fast‐spiking (FS) interneurons expressing parvalbumin (PV) are crucial components of DS pathophysiology.^10^, ^12^, ^13^, ^14^ PV + interneurons are hypoexcitable in *Scn1a^+/−^* mice, and sodium current (I_Na_) density is reduced in acutely isolated hippocampal interneurons.^9^, ^13^, ^15^ In contrast, data from mouse models have suggested that *Scn2a*‐, *Scn3a*‐, and *Scn8a*‐linked DEE variants are, in general, GOF, resulting in increased persistent or resurgent I_Na_ density and pyramidal neuron hyperexcitability.^5^, ^6^, ^7^, ^8^, ^16^ DEE treatment is informed by this dichotomy, for example, with VGSC blocking drugs contraindicated in *SCN1A*‐linked DS but recommended in *SCN8A*‐linked EIEE13.

EIEE52 (OMIM 617350) is a rare, early infantile DEE linked to autosomal recessive, bialleic, LOF variants in *SCN1B,* which encodes the VGSC β1/β1B subunits.^17^, ^18^ We reported that *SCN1B*‐linked DEE is, in general, more severe than *SCN1A*‐linked DS.^19^ VGSC β1 subunits associate with Nav1.1, Nav1.2, Nav1.3, and Nav1.6 and are expressed throughout the brain, raising the possibility that insults to both pyramidal and interneuron excitability may drive EIEE52 pathophysiology.^3^ *Scn1b* ^−/−^ mice model EIEE52, with spontaneous epilepsy and SUDEP in 100% of animals.^20^ Subpopulations of pyramidal neurons in *Scn1b* ^−/−^ or in the related *Scn1b‐*p.C121W knockin (*Scn1b^CW/CW^*) mouse model have higher AP firing rates than *Scn1b* ^+/+^, suggesting a disease mechanism that includes increased pyramidal neuron excitability.^21^, ^22^ However, contributions of *Scn1b* to PV + interneuron excitability, which have not been studied, may also be present, given the broad association of β1 with multiple VGSC α subunits. Here, we investigated excitability defects in cortical pyramidal neurons and PV + interneurons in the *Scn1b* ^‐/‐^ model of EIEE52. We also used *Scn1b^FL/FL^* mice ^23^ to delete *Scn1b* in specific neuronal populations. We found that *Scn1b* ^−/−^ cortical PV + interneurons are hypoexcitable, with reduced I_Na_ density. *Scn1b* ^−/−^ pyramidal neurons had population‐specific changes in excitability and I_Na_ density. These combined changes in excitatory and inhibitory neurons promote network hyperexcitability. *Scn1b* deletion in PV + neurons resulted in 100% lethality, whereas deletion in Emx1 + or Camk2a + neurons did not affect survival. Our results suggest that *SCN1B*‐linked DEE variants impact both excitatory and inhibitory neurons. This work may explain the increased severity of EIEE52 relative to other DEEs and inform the treatment of this devastating disease.

## Methods

### Animals

All experiments were performed according to NIH guidelines. Animal protocols were approved by the University of Michigan Institutional Animal Care and Use Committee. *Scn1b^‐/‐^* and *Scn1b^+/+^* littermate mice were generated as described^20^ and were congenic on the C57BL/6J background for over 20 N generations. *Scn1b^Fl/Fl^* mice, congenic on the C57BL/6J background, were generated as described.^23^ To generate mice with specific *Scn1b* deletion in labeled PV + neurons, *Scn1b^Fl/Fl^* mice were crossed with tdTomato (B6.129S6‐*Gt(ROSA)26Sor^tm14(CAG‐tdTomato)Hze^*/J, JAX #007908) and PV Cre (B6.129P2‐*Pvalb^tm1(cre)Arbr^*/J, JAX #017320) mice, both received from Dr. E. Goldberg at the University of Pennsylvania on the (C57BL/6J x CBA)F1 background and then backcrossed to C57BL/6J, to generate *Scn1b^Fl/Fl^*/*PV‐Cre/tdTom* and *Scn1b^E/E^*/*PV‐Cre/tdTom* mice. To label PV + FS neurons in *Scn1b^‐/‐^* brains, *Scn1b^+/‐^* mice were crossed with PV‐Cre/tdTom mice to generate *Scn1b^+/+^*/PV‐Cre/tdTom (*Scn1b* ^*+/+*^/PV) and *Scn1b^‐/‐^*/PV‐Cre/tdTom (*Scn1b* ^*‐/‐*^/PV) mice. *Scn1b* deletion in pyramidal neurons was achieved by crossing *Scn1b^Fl/Fl^* mice with Emx1‐Cre mice (B6.129S2‐Emx1^tm1^(cre)Krj/J, JAX 005628) or Camk2a‐Cre mice (B6.Cg‐Tg(Camk2a‐cre)T29‐1Stl/J, JAX 005359). Electrophysiological measurements were performed between postnatal day (P)14‐20.

### Immunohistochemistry

Coronal brain sections from P17‐P19 *Scn1b* ^+/+^/PV mice were generated at a thickness of 20 μm and labeled with anti‐PV antibodies as described.^24^ Sections were postfixed with 4% paraformaldehyde, washed with 0.05M phosphate buffer (PB), then blocked in PBTGS (0.1M PB, 0.3% Triton X‐100, 10% normal goat serum). Sections were incubated overnight in PBTGS containing rabbit anti‐PV antibodies (1:400, Abcam, cat. Ab11427). The following day, sections were washed with 0.1M PB, incubated with goat anti‐rabbit AlexaFluor647 in PBTGS, washed, and mounted with Prolong Gold. Images were acquired using a Nikon A1R confocal system with a Nikon FN1 microscope using a Nikon 20x 0.75 NA objective. Three fields of view per animal were analyzed using NIH ImageJ and assembled using Adobe Photoshop.

### Brain Slice Preparation

Acute brain slices were prepared as described.^21^ Mice were anesthetized with isoflurane anesthesia and decapitated. Brains were removed and placed in 95:5% O_2_:CO_2_ continuously aerated ice‐cold slice solution containing in mM: (110 sucrose; 62.5 NaCl; 2.5 KCl; 6 MgCl_2_; 1.25 KH_2_PO_4_; 26 NaHCO_3_; 0.5 CaCl_2_; and 20 D‐glucose (pH 7.35‐7.40 when aerated at RT). Brains were blocked and slices were obtained in 300‐μm‐thick coronal sections from primary sensory cortical areas (visual cortex for pyramidal neuron data for comparability to previous *Scn1b* model publications^21^) or somatosensory cortex (PV + neuron data for comparison to previous *Scn1a*‐DS publications^25^). Slices were incubated in an aerated holding chamber containing slice solution for 30 min at RT and then incubated in 1:1 slice:artificial cerebrospinal solution (ACSF) for 30 min. ACSF contained in mM (125 NaCl; 2.5 KCl; 1 MgCl_2_; 1.25 KH_2_PO_4_; 26 NaHCO_3_; 2 CaCl_2_; and 20 D‐glucose, pH 7.35‐7.40 with aeration). Slices were transferred to an aerated holding chamber containing 100% ACSF for at least 30 min before use.

### Action potential recording and analysis

Individual brain slices were placed in a recording chamber and superfused with 2–3 mL/min aerated ACSF. Pyramidal neurons were identified based on size, shape, and location using a Nikon A1R upright confocal microscope equipped with IR‐DIC optics with a 40X water immersion objective. Layer 5 was identified by large soma size and location, and layer 6 was identified as small pyramidal neurons between layer 5 and the boundary of the slice. Only vertically oriented pyramidal cells were selected for recording. FS interneurons were identified via red fluorescence using PV‐Cre/tdTom mice. Recording electrodes had a resistance of 3‐6 MΩ with solutions containing in mM (140 K‐Gluconate, 4 NaCl, 0.5 CaCl_2_, 10 HEPES, 5 EGTA, 5 phosphocreatine, 2 Mg‐ATP, and 0.4 GTP, pH adjusted to 7.2‐7.3 with KOH). The junction potential was calculated to be 14.3 mV using the P‐clamp junction potential calculator and all values were corrected offline, with all values presented in the study as corrected values. Following break‐in at −94.3 mV in voltage clamp mode, the resting membrane potential was defined as the membrane potential in current clamp < 10 s after initial break in. Repetitive firing was elicited in whole‐cell current clamp configuration from the resting membrane potential in 1‐s long current injections in 10 pA steps. There was a 1‐s long 0 current injection period between each sweep. Data were acquired at 20 kHz and were filtered at 10 kHz. Cells with an access resistance measured in voltage clamp> 20 MΩ or RMP>−64.3 mV were discarded. Access resistance and pipette capacitance were compensated using bridge balance. Whole‐cell capacitance was measured using P‐clamp whole‐cell capacitance compensation in voltage clamp with 10 mV depolarizing steps from −94.3 mV. Automated AP quantification was performed using custom MATLAB (MathWorks) software. APs were defined as the voltage crossing 0 mV subsequent to a dv/dt> 10 mV/ms, defined here as the AP threshold. Input resistance was calculated using Ohm’s law with −10 pA current injection from the resting membrane potential after 250 ms. After hyperpolarization (AHP) was defined as the difference between the minimum voltage reached after an action potential and the threshold of that action potential. Spike frequency adaptation was quantified as the ratio of the last interspike interval and the second interspike interval, measured at the maximum firing rate elicited during 1s long current injections.

### Neuronal dissociation and whole‐cell I_Na_ recording

Brain slices were prepared as above but maintained in 100% slice solution at RT until dissociation. Somatosensory cortex was isolated via microdissection using a 26.5‐gauge needle under a dissection microscope. Tissue was incubated at 35°C in oxygen‐saturated HBSS supplemented with 10 mM HEPES with 1.5 mg/ml protease type XIV (Sigma) for 23 min. Tissue was washed three times with oxygen‐saturated ice cold low‐calcium HBSS (1:10 HBSS with calcium and magnesium: HBSS calcium and magnesium Free) containing 10 mM HEPES. HBSS was replaced with ice cold, oxygen‐saturated Na‐isethionate solution (in mM; 140 Na‐isethionate, 23 glucose, 15 HEPES, 2KCl, 4MgCl2, 0.1 CaCl2) and tritiated with fire polished glass Pasteur pipettes to suspend cells. Cells were allowed to settle on a glass coverslip for 10 min prior to recording. All recordings were acquired < 1.5 h post dissociation. PV + cells were identified by red fluorescence and cells lacking neurites were not selected for recording. Voltage clamp recording was performed in the standard whole‐cell configuration with conditions described.^26^ Cells were superfused with external sodium channel recording solution containing in mM (30 NaCl, 1BaCl2, 2MgCl2, 45 CsCl, 0.2 CdCl2, 1CaCl2, 10 HEPES, 20 TEA‐Cl, and 100 D‐glucose, pH 7.35 with CsOH, Osmolarity 300–305 mOsm). Fire‐polished pipettes were filled with sodium channel internal recording solution containing in mM (115 CsCl, 0.5 CaCl_2_, 5 EGTA:CsOH, 10 HEPES, 5 Na_2_Phosphocreatine, 20 TEA, 2 Mg‐ATP, and 0.4 GTP, pH adjusted to 7.2‐7.3 with CsOH). The junction potential was calculated to be 2.7 mV with all reported voltages uncorrected.

### Calculation of permeability

To estimate the voltage dependence of activation for I_Na_ recordings, we calculated permeability using the Goldman–Hodgkin–Katz (GHK) current equation.

Equation 1:

$$I_{s}=P_{s}z_{s}^{2}\frac{EF^{2}}{\mathrm{RT}}\frac{{\left[S\right]}_{i}‐{\left[S\right]}_{o}\exp\left(‐\frac{z_{s}FE}{\mathrm{RT}}\right)}{1‐\exp\left(‐\frac{z_{s}FE}{\mathrm{RT}}\right)}$$

where *I_s_* is the measured current density, z_s_ is the ion valence, E is the membrane potential, F is Faraday’s constant, [S]_i_ and [S]_o_ are the internal and external ion concentrations, respectively, and P_s_ is the calculated permeability.^27^ Data were calculated by fitting with a Boltzmann equation for the calculation of the voltage dependence of activation and inactivation.

### sIPSC Recording

sIPSCs were recorded under similar slice conditions as AP recordings, altered to allow for recording of sIPSCs. Intracellular solution contained (in mM): CsCl (135), NaCl (4), GTP (0.4), Mg‐ATP (2), CaCl2 (0.5), EGTA (5), and HEPES (10). Currents were recorded in voltage clamp at a holding potential of −80 mV after> 5 min in the whole‐cell configuration. Our laboratory previously measured, using the perforated patch technique, a depolarized reversal potential of synaptic Cl^‐^ currents in *Scn1b^‐/‐^* mice relative to their WT littermates.^28^ To ensure that this abnormal intracellular Cl^‐^ concentration did not affect our synaptic recordings in this study, we used the whole‐cell recording technique with high intracellular Cl^‐^ to abolish any effects on the detected frequency of events. We compared the cumulative probability distributions of event amplitudes between genotypes and found that, following the 5‐min equilibration period with the whole‐cell internal solution, that they were not from statistically different distributions using the KS test (Fig. 6B), indicating this manipulation was sufficient to washout any effects that an altered Cl^‐^ reversal potential may have had on event amplitudes and, by association, event detection. CNQX (10 µmol/L) and APV (50 µmol/L) were applied for> 5 min to block excitatory synaptic currents. sIPSCS were recorded for 5 min at a sampling rate of 20 kHz and filtered at 2kHz. sIPSCs were autonomously analyzed using MiniAnalysis software with a 10 pA amplitude threshold and visual exclusion/inclusion of events. Mean frequency and amplitude were calculated from the averages of all events for each cell recorded over the 5‐min period. Cumulative probability distributions were calculated by sampling 100 random events from each cell and combining across cells to compare conditions.

### Nucleated patch clamp I_Na_ recording

Nucleated patches were pulled from intact layer 6 pyramidal neurons in acute brain slices. Internal solution was as above for whole‐cell I_Na_ recordings. The junction potential was calculated to be 3.5 mV and all values presented are corrected. Patches were pulled by applying light suction in the whole‐cell configuration while slowly withdrawing the pipet over 5–10 min. Suction was then removed before recording. External recording solution consisted of aerated ACSF with 200 μmol/L Cd^2+^ added after establishment of the whole‐cell configuration. Slices were discarded if perfused with Cd^2+^ >60s to maintain slice health. Patches with access resistance> 20 MΩ or with input resistance < 1 GΩ were discarded.

### Persistent I_Na_ recording

Persistent I_Na_ in brain slices was measured in the whole‐cell configuration in ACSF supplemented with 200 µmol/L CdCl_2_. Recordings were attained with the same internal solution as for whole‐cell I_Na_ recordings. Currents were elicited using a slow voltage ramp (150 mV/3s) after> 5 min following initial break in. Any residual transient spikes were removed using a linear interpolation between points before and after the transient. Four trials were averaged for each cell and current density was calculated at −20 mV.

### Statistics

No more than 1 cell from a brain region was acquired per slice, no more than four cells per region were acquired per animal, and each experiment was performed with at least three animals. Each cell is reported as *n* = 1 and each animal is reported as *N* = 1. Significance was set at *P* < 0.05. Comparisons were made with an unpaired two‐tailed Student’s t‐test unless stated otherwise in the text. All listed *P*‐values are tested for significance after multiple comparisons and reported as nonsignificant if *q* > 0.05 using the Benjamini, Krieger, and Yekutieli two stage step‐up method with a 5% false discovery rate correction. Values were tested for a non‐Gaussian distribution using D’Agostino‐Pearson omnibus normality test. Differences in variance were tested using an F‐Test.

## Results

### *Scn1b* deletion in PV + neurons results in seizures and SUDEP

**Figure 1:**
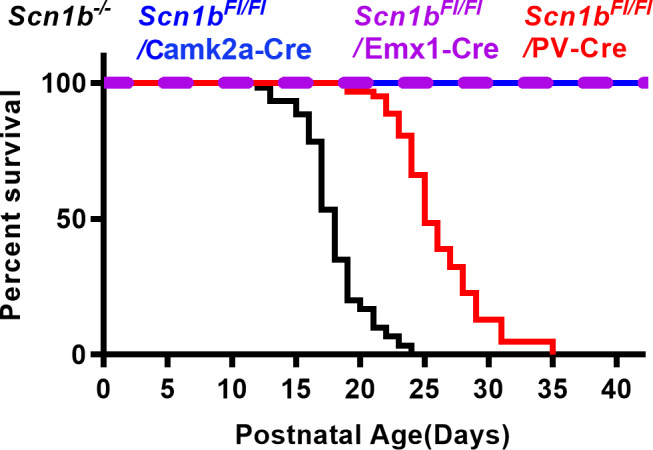
PV‐dependent Cre deletion of *Scn1b* results in early lethality while Emx1 and or Camk2a‐dependent Cre deletion does not. A. Kaplan–Meier survival curve of Scn1b*^Fl^* ^/^ *^Fl^*/PV‐Cre mice (median 25 days, *N* = 62) compared to *Scn1b* ^−/−^ mice (median of 18 days, *N* = 60, *P* < 0.0001, Mantel–Cox test), *Scn1b^Fl/Fl^*/CamK2a‐Cre and *Scn1b^Fl/Fl^*/Emx1‐Cre mice, (no deaths, *N* = 21 and *N* = 15, respectively, both *P* < 0.0001, Mantel–Cox test).

To examine the roles of PV + vs. excitatory neurons in *Scn1b*‐linked DEE pathology, we generated *Scn1b^Fl/Fl^*/PV‐Cre mice, *Scn1b^Fl/Fl^*/Emx1‐Cre mice, and *Scn1b^Fl/Fl^*/ Camk2a‐Cre mice. Selective *Scn1b* deletion in PV + neurons resulted in epilepsy and 100% lethality, with disease onset displaced by one week relative to *Scn1b* ^‐/‐^ mice. Median survival was 18 days for *Scn1b* ^−/−^ mice and 25 days for *Scn1b^Fl/Fl^*/PV‐Cre mice (Fig. 1A, *P* < 0.0001, Mantel–Cox test). Movie S1 shows an example of a spontaneous convulsive seizure in a P17 *Scn1b^Fl/Fl^*/PV‐Cre mouse. The failure‐to‐thrive phenotype of *Scn1b^‐/‐^* pups precluded the implantation of EEG electrodes.^28^ Behavioral seizures were not observed in *Scn1b^Fl/Fl^*/Emx1‐Cre or *Scn1b^Fl/Fl^*/ Camk2a‐Cre mice and all mice survived for more than 90 days. These data suggest a crucial role for PV + interneurons in *Scn1b*‐linked seizures and SUDEP.

### *Scn1b* deletion results in PV + cortical interneuron hypoexcitability

**Figure 2:**
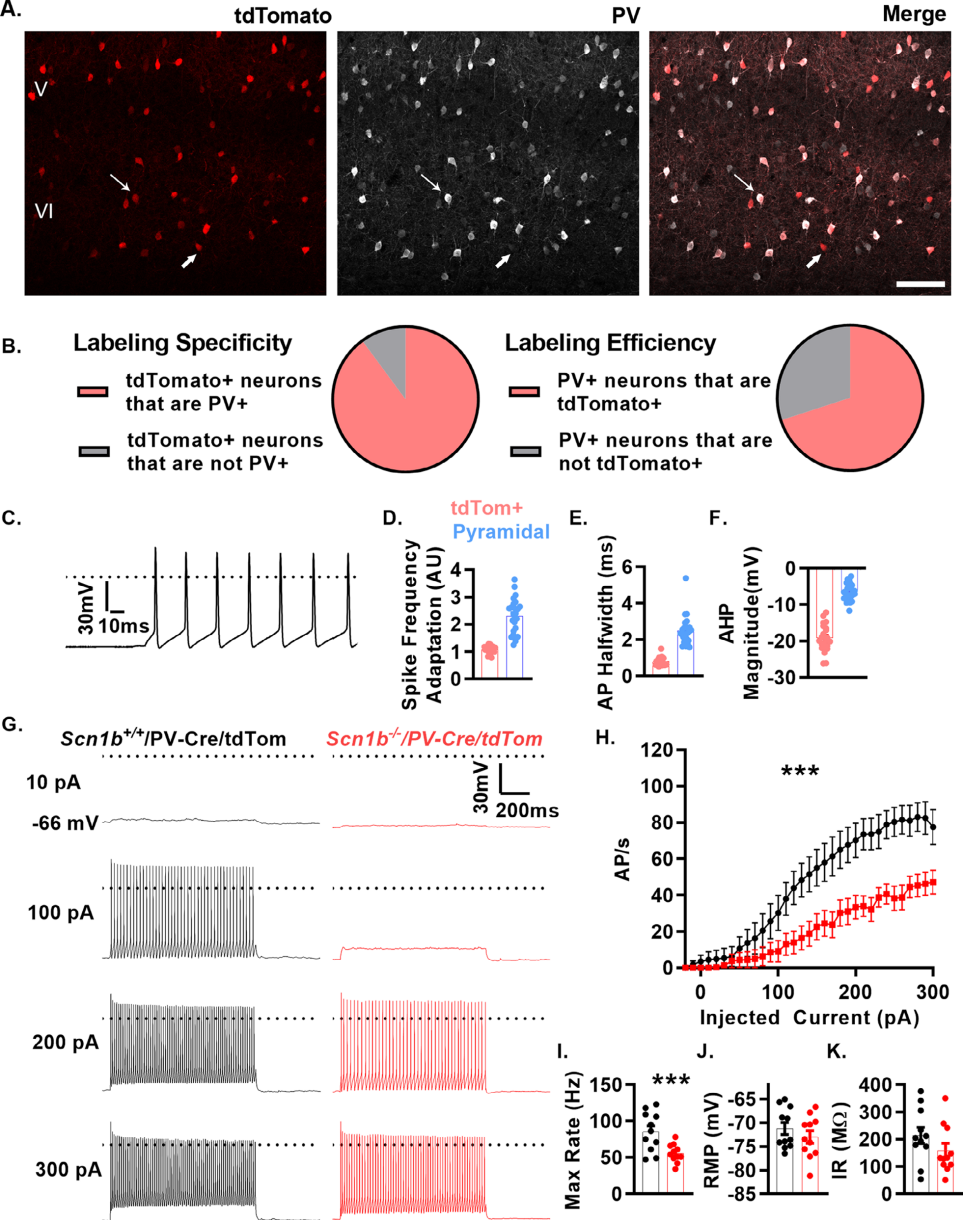
*Scn1b* deletion results in cortical PV + neuron hypoexcitability. (A) Representative images of tdTom + cells (left), anti‐PV labeling (middle), and merge (right). Most tdTom + cells (89%) are also detected with anti‐PV antibodies (upper arrow). Few tdTom + neurons display minimal to no labeling with anti‐PV antibodies (lower arrow). Scale bar = 100 μm. (B) Pie charts demonstrating the extent of labeling specificity (left) and labeling efficiency (right). (C) Representative voltage traces from whole‐cell recordings of neurons positive for red fluorescence (n/N = 22/8) from brain slices of *Scn1*b^+/+^/PV‐Cre/tdTom (*Scn1b* ^+/+^/PV in text) or *Scn1b* ^‐/‐^/PV‐Cre/tdTom (*Scn1b* ^‐/‐^/PV in text) mice at P14‐20. (D–F) Characteristic features of FS interneurons were present in all tdTom‐labeled neurons recorded relative to layer 5 pyramidal neurons (see Fig. 4 for additional details) including low spike frequency adaptation (D.), short AP half width (E.), and large AHP magnitudes (F). (G) Representative voltage traces of *Scn1b* ^+/+^/PV (black) or *Scn1b* ^‐/‐^/PV mice (red). (H) Current injection vs. APs fired in 1‐s of recordings above. Asterisks indicate *P* value for area under the curve (***P < 0.005, n/N = 11/4 *Scn1b* ^+/+^/PV 11/4 *Scn1b* ^‐/‐^/PV. (E) Maximum firing rate for neurons in B (***P < 0.005). (I‐K) Average resting membrane potential (F) and input resistance (G) from recordings in G. See Table 1 for numerical data and biophysical properties.

To test whether PV + interneuron excitability is altered by *Scn1b* deletion, we intercrossed *Scn1b* ^+/−^ mice with a strain expressing the tdTomato/Ai14 fluorescent reporter under control of the PV promoter, generating *Scn1b* ^+/+^/PV and *Scn1b* ^−/−^/PV mice, to facilitate the identification of PV + neurons in brain slices via epifluorescence. Figure 2A shows the extent of expression of tdTomato (tdTom) reporter expression and co‐localization with anti‐PV antibody staining, demonstrating a high degree of PV‐positive (+) neuron specificity for tdTom expression (89.94 +/− 3.18% of tdTom + cells were PV+, Fig. 2B left) and a high labeling efficiency (69.98 +/− 3.15% of PV + cells were tdTom+, Fig. 2B right). We recorded APs from tdTom + cells in acute *Scn1b* ^‐/‐^/PV or *Scn1b* ^+/+^/PV cortical layer 5 brain slices with 1‐s long current injections ranging from −20 to 300 pA (Fig. 2C) to measure FS neuron excitability. All recorded cells showed features predictive of FS interneurons, including nonaccommodating APs (average: 1.09+/‐ 0.03 AU), narrow AP half‐widths (0.79 ± 0.05 ms), and large AHP magnitudes (average: −19.2 ± 0.83 mV) (Fig. 2C‐F). For comparison, these data were plotted alongside our results for layer 5 pyramidal neurons (shown below in Fig. 4A‐D), demonstrating nearly complete lack of overlap between tdTom + FS features from those of non‐FS pyramidal neurons. The maximal firing frequency was significantly reduced in *Scn1b^‐/‐^/*PV neurons relative to *Scn1b^+/+^*/PV littermates. (Fig. 2H). Fig. 2G shows representative traces comparing *Scn1b* ^‐/‐^/PV and *Scn1b* ^+/+^/PV firing patterns at 10, 100, 200, and 300 pA. We found no changes in input resistance (Fig. 2K, Table 1), whole‐cell capacitance (Table 1), or resting membrane potential (RMP) between genotypes (Fig. 2J, Table 1). We compared AP peak voltage, peak dv/dt, and AP voltage threshold in AP trains elicited by 1‐s long current injections from the recordings in Fig. 2H and Table 1 at 20 pA above the threshold to fire repetitive (>3) APs. Neither the first AP nor subsequent APs in the train were different between genotypes in AP peak voltage, however, repetitive firing revealed a decrease in the peak dv/dt and higher AP threshold in the *Scn1b* ^‐/‐^/PV neurons compared to *Scn1b* ^+/+^/PV (Table 1).

**Table 1:** *Scn1b* deletion alters excitability of PV + interneurons and pyramidal neurons.

		PV‐Cre/tdTom+	Layer 5	Subiculum	Layer 6
	*Scn1b:*				
Resting V_m_	+/+	−71.2 ± 1.3	−84.7 ± 1.2	−78.5 ± 0.5*	−81.7 ± 1.2*
(mV)	−/−	−73.0 ± 1.3	−84.6 ± 1.7	−75.5 ± 1.0	−76.8 ± 1.5
Input Resistance	+/+	214.2 ± 29.8	91.7 ± 5.6	135.7 ± 53.5***	461.6 ± 36.4****
(MΩ)	−/−	159.1 ± 26.6	101.5 ± 4.9	247.0 ± 29.7	669.4 ± 37.4
Capacitance	+/+	21.7 ± 3.0	52.2 ± 3.4	32.8 ± 2.3	19.5 ± 2.1***
	−/−	22.0 ± 1.4	43.2 ± 2.2	27.5 ± 1.7	14.3 ± 1.0
Max Firing Rate	+/+	85.5 ± 8.1***	22.2 ± 1.3	26.9 ± 1.4	24.8 ± 1.2
	−/−	55.6 ± 3.7	19.6 ± 1.0	26.6 ± 1.1	22.9 ± 1.0
Depolarization Block	+/+	10.5 ± 6.9	49.6 ± 12.2	22.4 ± 7.0(*)	63.7 ± 9.3^***^
(%)	−/−	15.7 ± 10.3	47.8 ± 12.9	46.5 ± 9.2	92.8 ± 1.1
1^st^ AP Peak Voltage	+/+	18.5 ± 3.6	22.0 ± 1.3	30.7 ± 1.2	24.8 ± 1.0
(mV)	−/−	14.8 ± 3.1	23.2 ± 1.2	27.6 ± 1.5	22.9 ± 0.9
AP Train Peak Voltage	+/+	14.3 ± 3.4	12.1 ± 2.1	24.6 ± 1.4**	18.0 ± 1.3****
(mV)	−/−	9.1 ± 3.2	15.2 ± 1.8	18.0 ± 1.9	9.1 ± 1.1
1^st^ AP Peak dv/dt	+/+	272.8 ± 23.4	176.3 ± 8.4	286.7 ± 11.4***	170.1 ± 6.5
(mV/ms)	−/−	209.6 ± 27.2	187.3 ± 5.9	231.3 ± 13.3	158.0 ± 6.5
AP Peak dv/dt Train	+/+	223.5 ± 21.4*	111.0 ± 10.4	203.0 ± 14.6***	116.2.2 ± 8.2***
(mV/ms)	−/−	156.6 ± 20.0	134.7 ± 8.5	131.5 ± 13.3	73.4.3 ± 7.3
1^st^ AP Threshold	+/+	−60.1 ± 1.5	−68.7 ± 0.9	−68.7 ± 0.8	−65.2 ± 0.7
(mV)	−/−	‐60.8 ± 2.8	−68.4 ± 1.0	−67.8 ± 1.1	−64.3 ± 0.8
AP Threshold Train	+/+	−55.5 ± 0.9*	−60.9 ± 0.9	−60.8 ± 0.9**	−56.5 ± 0.9***
(mV)	−/−	‐52.9 ± 0.9	62.2 ± 1.5	−56.9 ± 1.1	−51.6 ± 1.2

Data shown graphically in Fig. 2 and 4. Values are mean ± SEM.

^1^ Indicates t‐test with Welch’s Correction.

* *P* < 0.05

** *P* < 0.01

*** *P* < 0.005,

**** *P* < 0.0001

### I_Na_ is reduced in dissociated *Scn1b^‐/‐^* cortical PV + interneurons

**Figure 3:**
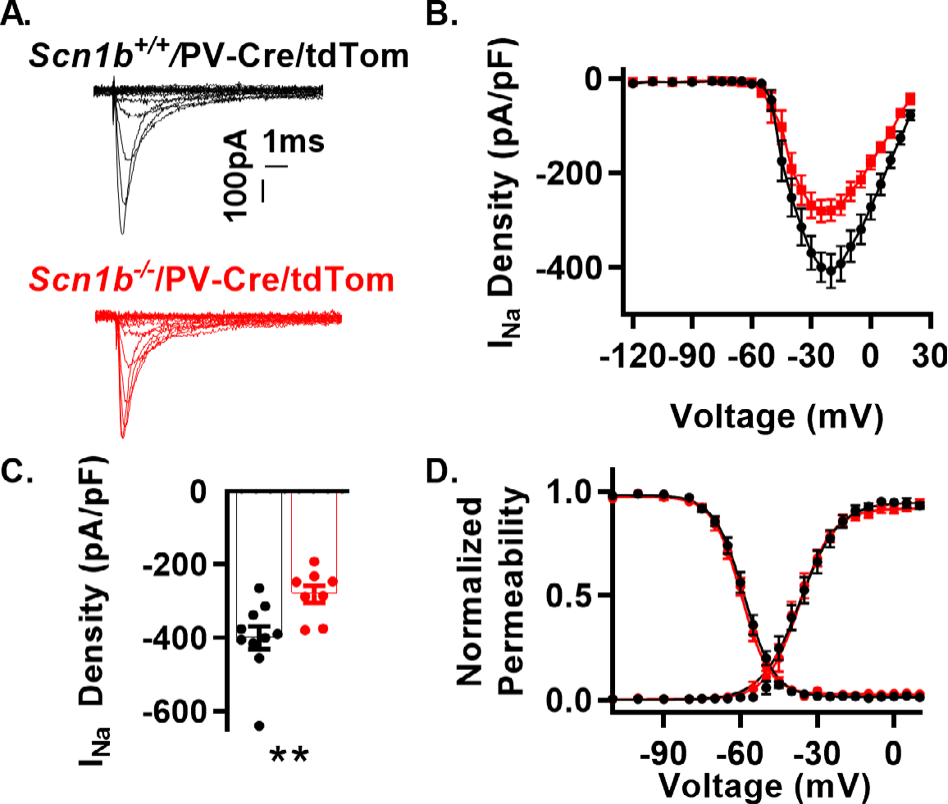
*Scn1b* deletion reduces I_Na_ density in dissociated cortical PV + neurons (A) Representative I_Na_ traces from whole‐cell recordings from cortical tdTom + neurons of *Scn1b* ^+/+^/PV and *Scn1b* ^‐/‐^/PV mice. Current elicited by depolarizing steps from −120 mV to 30 mV from a holding potential of −120 mV (traces up to peak are shown for visualization of smaller nested currents). (B) Current–voltage relationship for recordings as in A (n/N = 10/3 *Scn1b* ^+/+^/PV, 8/3 *Scn1b* ^‐/‐^/PV). (C) Peak I_Na_ density at −25 mV from recordings in B. Asterisks indicate *P* value (**P < 0.01). (D) Normalized voltage dependence of steady‐state activation and inactivation of recordings in B. See Table 2 for numerical values

We recorded I_Na_ in acutely dissociated tdTom + cortical interneurons of *Scn1b^‐/‐^*/PV and *Scn1b^+/+^*/PV mice in cells identified by red epifluorescence (Fig. 3A). I_Na_ density at the mean peak current density (−25 mV) was reduced by approximately 30% in *Scn1b^−/−^* interneurons compared to *Scn1b^+/+^* (Fig. 3B,C and Table 2). The V_1/2_ and slope factors for voltage‐dependent permeability and inactivation were unaffected by *Scn1b* deletion (Fig. 3D and Table 2).

**Table 2:** Effects of *Scn1b* deletion on I_Na_ in PV + interneurons and pyramidal neurons.

		Dissociated PV−Cre/tdTom+ Interneurons	Layer 6 Pyramidal Neurons Nucleated Patches	Layer 6 Pyramidal Neuron Voltage Ramps
	*Scn1b:*			
Peak Current Density	+/+	−399.52 ± 31.78**	−218.33 ± 36.33^1^, *	−10.30 ± 0.71***
(pA/pF)	−/−	−280.39 ± 23.50	−134.36 ± 4.30	−6.70 ± 0.54
Capacitance (pF)	+/+	7.52 ± 0.47	1.23 ± 0.07	20.90 ± 1.33*
	−/−	8.36 ± 0.55	1.16 ± 0.05	16.63 ± 1.12
Activation V_1/2_ (mV)	+/+	−35.45 ± 1.70	−38.75 ± 1.69	−47.70 ± 1.70
	−/−	−37.83 ± 2.27	−36.52 ± 1.94	−46.86 ± 1.09
Activation k (mV)^−1^	+/+	6.47 ± 0.52	6.03 ± 0.57	5.54 ± 0.33
	−/−	5.86 ± 0.29	6.51 ± 0.45	5.89 ± 0.42
Inactivation V_1/2_ (mv)	+/+	−58.56 ± 1.22	−67.10 ± 1.43	N.A.
	−/−	−59.36 ± 0.97	−71.11 ± 2.43	N.A.
Inactivation k (mV)^−1^	+/+	5.30 ± 0.11	5.2 ± 0.37*	N.A.
	−/−	5.28 ± 0.10	6.86 ± 0.49	N.A.

Data shown graphically in Fig. 3 and 5. Values are mean ± SEM.

^1^ indicates t‐test with Welch’s Correction

* *P* < 0.05

** *P* < 0.01

*** *P* < 0.005

### Subpopulations of *Scn1b^‐/‐^* pyramidal neurons show complex excitability phenotypes

**Figure 4:**
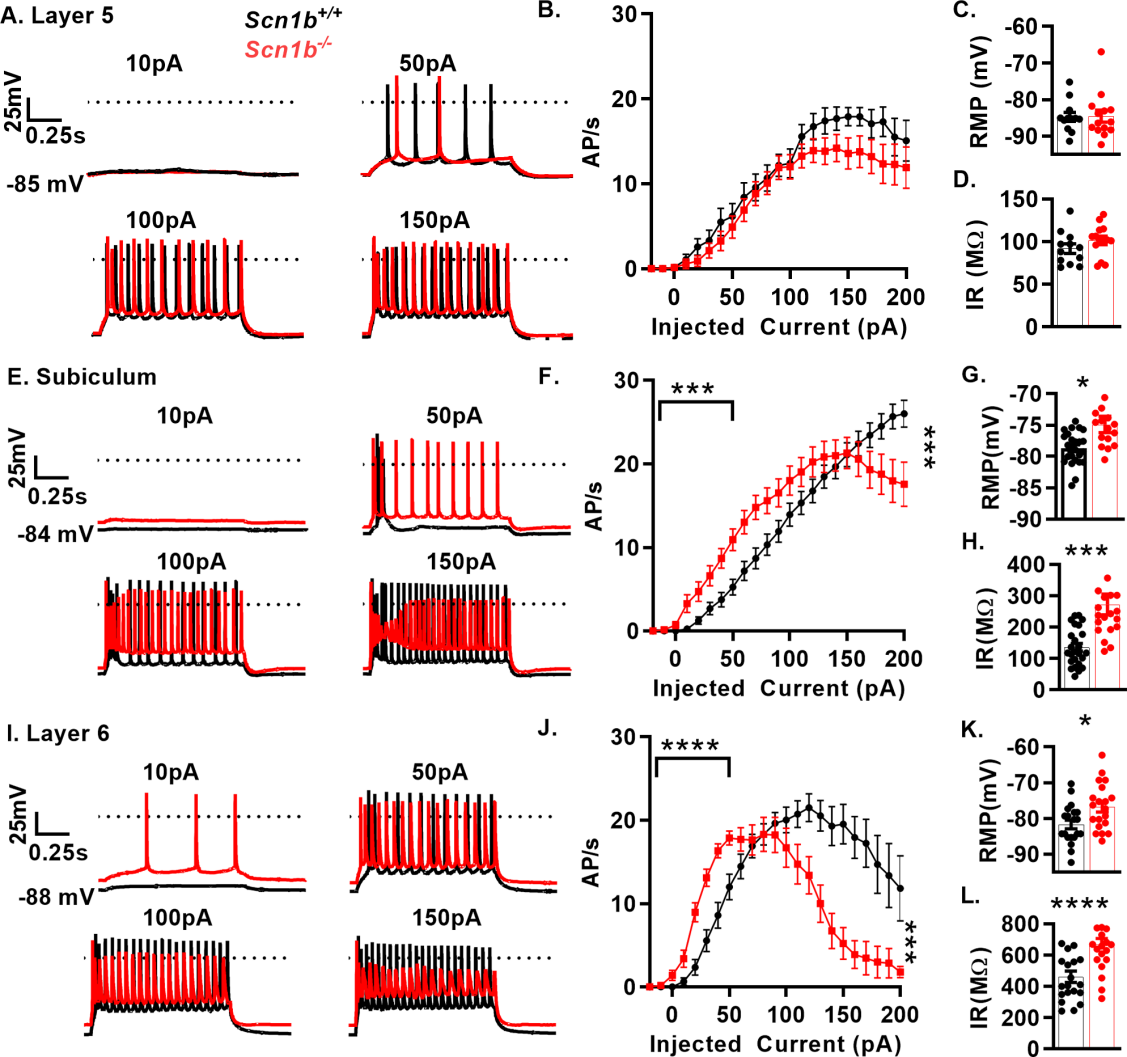
*Scn1b* deletion results in complex pyramidal neuron excitability defects. A., E, and I. Representative voltage traces from whole‐cell recordings of layer 5 (A), subiculum (E) and layer 6 (I) pyramidal neurons in acute brain slices of *Scn1b* ^+/+^ (black) or *Scn1b* ^‐/‐^ mice (red) B., F., J., Current injection vs. APs fired in 1‐s of recordings above. Firing at low current injection quantified as area under the curve up to 50 pA. Depolarization block is quantified as AP count at highest current injection divided by max AP count (with AP failure defined as max voltage < 0 mV, right asterisks). Layer 5 (B) pyramidal neurons show no change in firing at low current injections or degree of depolarization block (n/N = 12/7 *Scn1b* ^+/+^, 14/7 *Scn1b* ^‐/‐^). Subicular (F) and Layer 6 pyramidal neurons (J) and show increased firing at low current amplitudes (left asterisks indicate p‐value) in *Scn1b* ^+/+^ vs. *Scn1b* ^‐/‐^ mice (n/N = 25/12 *Scn1b^+/+^*, 24/13 *Scn1b^‐/‐^* subiculum; n/N = 20/10 *Scn1b* ^+/+^, 21/9 *Scn1b* ^‐/‐^ layer 6, Welch’s t‐test). C., G., K. RMP is not affected by *Scn1b* deletion in layer 5 (C) but is depolarized in subicular (G) and layer 6 pyramidal neurons (K). D., H., L. Input resistance is unaffected by *Scn1b* deletion in layer 5 (D) but is increased in subicular (H) and layer 6 pyramidal neurons (L). See Table 3 for quantification of biophysical properties. Asterisks indicate p‐values (*P < 0.05, ***P < 0.005, and ****P < 0.0001)

Previous work has shown *Scn1b^−/−^* and *Scn1b^CW/CW^* pyramidal neuron hyperexcitability relative to *Scn1b* ^+/+^ neurons, but with variable, population‐dependent results.^21^, ^22^ Here, we recorded from several pyramidal neuron populations to determine whether *Scn1b* deletion resulted in excitability defects. APs were recorded in 1‐s long current injections ranging from −20 to 200 pA, from pyramidal neurons in cortical layers 5 and 6 and the subiculum in acute brain slices from *Scn1b^‐/‐^* and *Scn1b^+/+^* littermate mice. We found no differences in evoked firing rates between genotypes in layer 5 pyramidal neurons (Fig. 4, panels A and B). In contrast, we observed altered excitability in *Scn1b^‐/‐^* subiculum (Fig. 4, panels E and F) and layer 6 (Fig. 4, panels I and J) neurons compared to *Scn1b^+/+^*.

Fig. 4, panels A, E, and I show representative traces comparing P14‐20 *Scn1b^−/−^* and *Scn1b^+/+^* cortical layer 5, subiculum, and layer 6 pyramidal neuron firing patterns at 10, 50, 100, and 150 pA. In layer 5, neurons showed no change in RMP, capacitance, or input resistance, whereas in subiculum and layer 6, *Scn1b^‐/‐^* neurons showed depolarized RMP, increased input resistance, and decreased capacitance in layer 6 (Fig. 4, panels C, D, G, H, K, and L and Table 1).

Increased firing in *Scn1b^‐/‐^* subicular and layer 6 neurons was limited to low current injections (Fig. 4F and J, respectively), quantified as area under the curve up to 50 pA of injected current. To quantify differential sensitivity of neurons to depolarization block at higher current injections, firing rate at the highest current injection was divided by the maximum firing rate for that cell. *Scn1b^‐/‐^* layer 6 neurons showed a greater degree of attenuation compared to *Scn1b^+/+^*, with subiculum showing similar results (Table 1). We compared AP peak voltage, peak dv/dt, and AP voltage threshold between genotypes for neurons in layers 5, 6 and subiculum in AP trains elicited by 1‐s long current injections from the recordings shown above. We analyzed layer 6 at 100 pA and subiculum and layer 5 at 150 pA due to near total depolarization block at 150 pA in layer 6 and the lower firing rate at 100 pA in layer 5 and subiculum. Both layer 6 and subicular pyramidal neurons exhibit alterations in several VGSC related AP parameters (Table 1). Pyramidal neurons in *Scn1b^‐/‐^* cortical layer 5 were the least affected population examined, with no difference between genotypes in AP properties (Table 1). These results show that, in addition to FS interneuron hypoexcitability shown in Fig. 2, pyramidal neurons are affected by *Scn1b* deletion, with subsets of pyramidal neurons exhibiting hyperexcitability at low current injections as well as hypoexcitability at high stimulation intensities.

### *Scn1b* deletion reduces transient and persistent I_Na_ density in layer 6 pyramidal neurons

**Figure 5:**
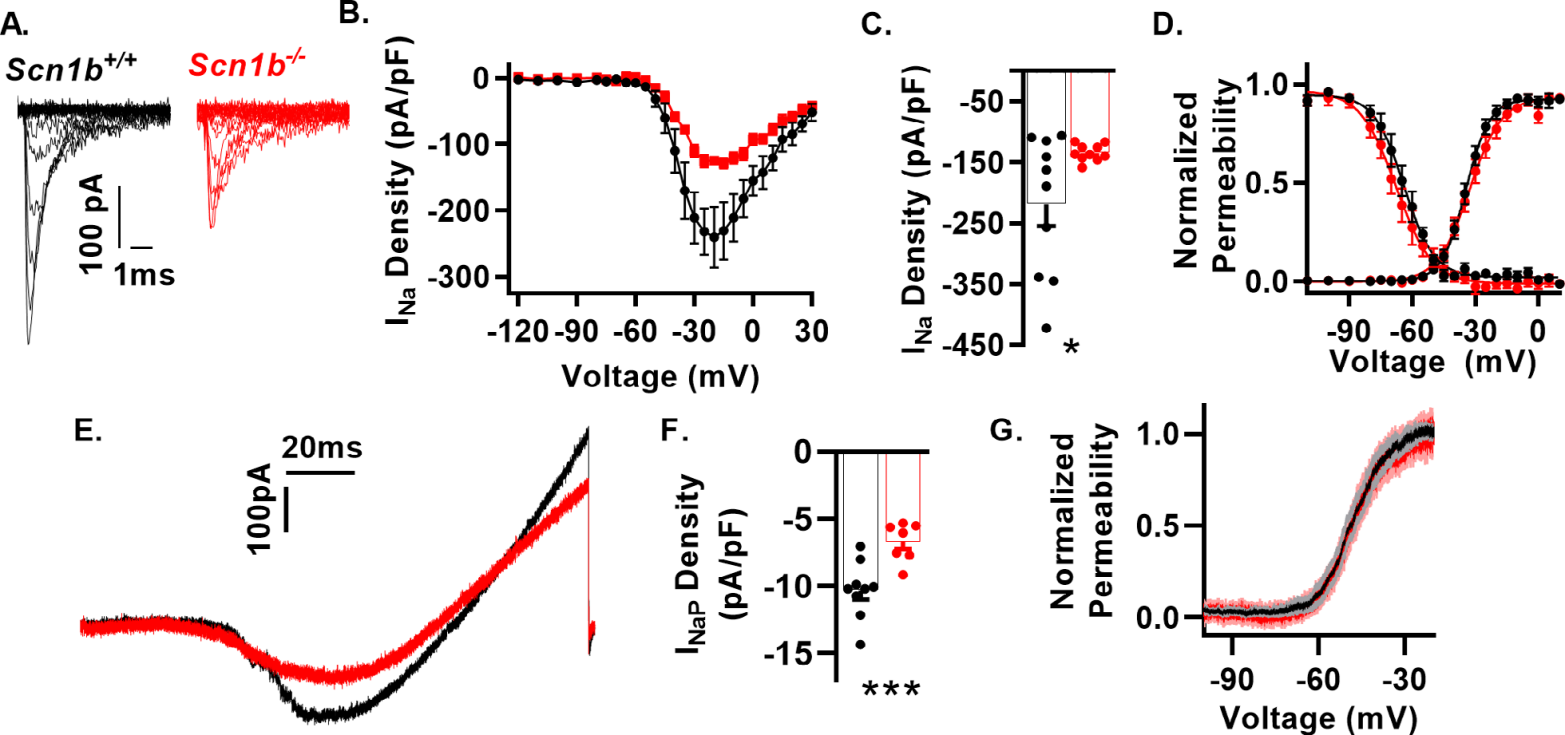
*Scn1b* deletion results in reduced transient and persistent I_Na_ in layer 6 pyramidal neurons. (A) Representative I_Na_ traces from nucleated patches from cortical layer 6 pyramidal neurons of *Scn1b* ^+/+^ and *Scn1b* ^−/−^ mice in acute brain slices. Current elicited by depolarizing steps from −120 mV to 30 mV from a holding potential of −120 mV. (B) Current–voltage relationship for nucleated patches as in A (n/N = 10/10 *Scn1b* ^+/+^, 10/9 *Scn1b* ^−/−^). (C) Peak I_Na_ density at −20 mV from recordings in B. Welch’s t‐test. Asterisks indicate *P* value (*P < 0.05). (D) Normalized voltage dependence of steady state activation and inactivation of recordings in B. (E) Representative persistent I_Na_ recorded in intact neurons in *Scn1b* ^+/+^ and *Scn1b* ^−/−^ cortical layer 6 pyramidal neurons in acute brain slices using a 150 mV/3s voltage ramp starting from a holding potential of −120 mV. Traces are the average of four recordings from the same neuron. F. Persistent I_Na_ at −20 mV (n/N = 9/4 *Scn1b* ^+/+^, 7/3 *Scn1b* ^‐/‐^). (G) Voltage dependence of activation for data in F. Asterisks indicate *P* value (*P < 0.05 and ***P < 0.005).

We recorded I_Na_ using nucleated patches from pyramidal neurons in *Scn1b* ^‐/‐^ visual cortex layer 6, a hyperexcitable population identified in Fig. 5. I_Na_ density at −20 mV was reduced approximately 40% in *Scn1b* ^−/−^ layer 6 nucleated patches compared to *Scn1b* ^+/+^ (Fig. 5, panels A, B, C and Table 2). We observed no changes in the voltage dependence of activation or inactivation with the exception of a decrease in k of inactivation. Because changes in persistent I_Na_ are of special relevance to understanding other DEE models,^29^ we recorded persistent I_Na_ in intact pyramidal neurons of layer 6 brain slices, and found it to be decreased compared to *Scn1b^+/+^*, to a similar degree as transient I_Na_ in nucleated patches (Fig. 5F). We observed no changes in the voltage dependence of activation between genotypes (Fig. 5G). Similar to the AP recordings, we observed a decrease in whole‐cell capacitance in the null neurons (Table 2). Together, these results suggest that pyramidal neuron hyperexcitability in *Scn1b* ^−/−^ mice is not due to increased persistent I_Na_, as observed with VGSC GOF variants, but instead may be attributable to previously reported effects on I_K_ or decreased cell size as recorded here.^22^, ^30^

### *Scn1b^−/−^* pyramidal neurons are disinhibited

**Figure 6:**
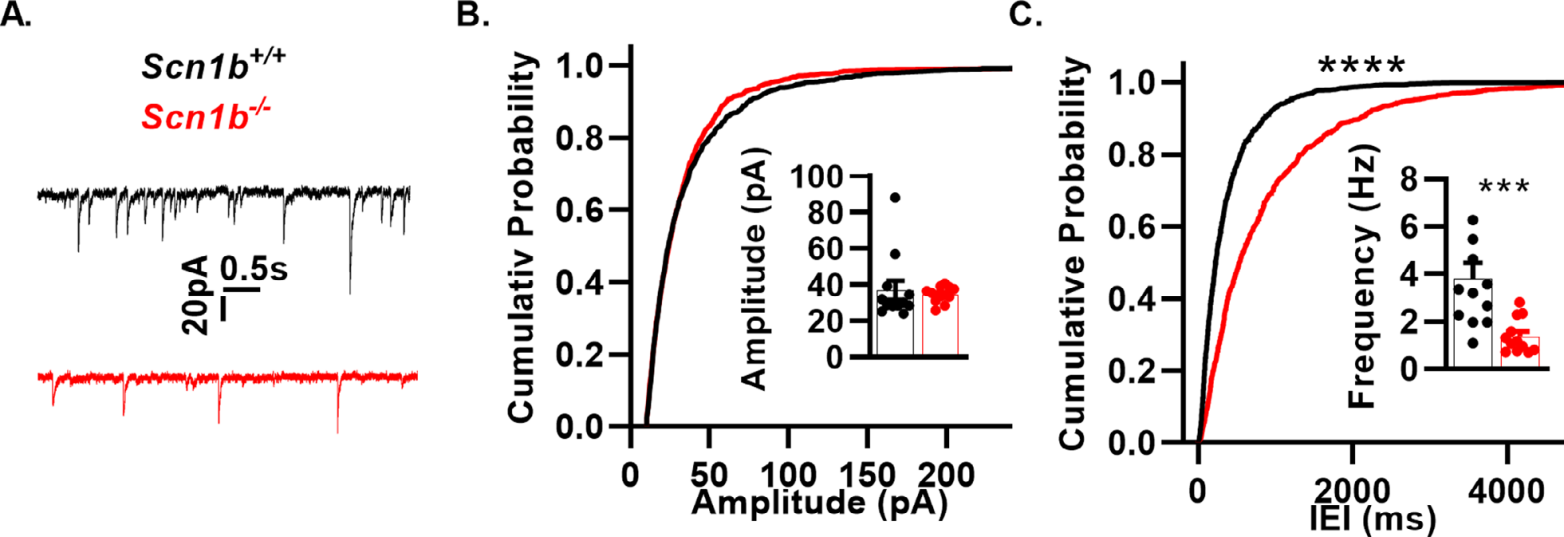
*Scn1b* deletion impairs inhibitory neurotransmission. (A) Representative sIPSC traces from whole‐cell recordings from layer 6 pyramidal neurons of *Scn1b* ^+/+^ (black) and *Scn1b* ^‐/‐^ (red) mice. (B) Cumulative probability plots of sIPSC amplitude (n/N = 12/4 *Scn1b* ^+/+^, 12/4 *Scn1b* ^‐/‐^) as in A. Inset bar graphs show mean sIPSC amplitude. (C) Cumulative probability plot of interevent intervals as in A. Top asterisks indicate *P*‐value for comparing cumulative probabilities (****P < 0.0001, Kolmogorov–Smirnov test). Inset bar graphs shown mean sIPSC frequency (1/IEI). Asterisk indicates *P*‐value (***P < 0.005, Welch’s t‐test).

Previous work showed *Scn1b* ^‐/‐^ cortical network hyperexcitability, however, impairments in inhibitory neurotransmission were not investigated.^21^ To test for these changes, we recorded sIPSCs in *Scn1b^+/+^* and *Scn1b^−/−^* brain layer 6 cortical pyramidal neurons. *Scn1b^−/−^* pyramidal neurons showed no change in the average amplitude (34.54 ± 1.28 pA in *Scn1b^−/−^*, n/N = 12/4 vs. 36.90 ± 5.28 pA in *Scn1b^+/+^*, N = 12/4) or cumulative probability distribution of current amplitudes compared to *Scn1b^+/+^*, but received a 2.76 fold lower average frequency (1.38 ± 0.21 Hz in *Scn1b^−/−^*, n/N = 12/4 vs. 3.81 ± 0.66 Hz in *Scn1b^+/+^*, *N* = 12/4, *P* < 0.005, Welch’s t‐test, *F* = 9.98, *P* < 0.005, F‐test) of sIPSCs (Fig. 6F) accompanied by an altered cumulative probability distribution, showing that *Scn1b* deletion results in impairments to inhibitory neurotransmission.

## Discussion

Early infantile DEE resulting from homozygous *SCN1B* LOF variants has a more severe clinical phenotype, with earlier onset, than typical DS.^19^ Here, we used *Scn1b* mouse models to investigate changes in neuronal excitability underlying EIEE52. We show that PV + neuron specific *Scn1b* deletion using a Cre‐lox strategy is sufficient to cause seizures and SUDEP, suggesting that this neuronal population contributes significantly to *Scn1b* ^‐/‐^ pathology. In contrast, two different models of excitatory neuron specific *Scn1b* deletion showed no changes in life span or incidence of behavioral seizures, suggesting that changes in pyramidal neuron excitability in *Scn1b^‐/‐^* cortical layer 6 may contribute to other co‐morbidities associated with EIEE52. Subpopulations of cortical *Scn1b^‐/‐^* pyramidal neurons were hyperexcitable at low current injections (at the firing rates most relevant *in vivo*) but had early depolarization block. The importance of layer 6 and subicular hyperexcitability at these lower current injections is highly relevant for generalized epilepsy, where the hyperexcitability of circuit outputs are likely to contribute to the distribution of seizure activity across wide ranging brain areas. Transient and persistent I_Na_ densities were decreased in *Scn1b* ^‐/‐^ layer 6 pyramidal neurons, suggesting that alternative mechanisms of pyramidal neuron hyperexcitability, such altered I_K_ or cell size, may play critical roles.^22^, ^30^ Future work may show that alterations to I_K_ in interneurons are yet another crucial component to disease manifestation that may exacerbate the I_Na_ defects measured here. Importantly, changes in the excitability of both pyramidal and interneurons in *Scn1b^−/−^* brain suggest that therapeutic efficacy or safety may diverge significantly in EIEE52 patients from those with *SCN1A* or *SCN8A* DEE variants.

Selective *Scn1b* deletion in PV + neurons was sufficient to generate spontaneous convulsive seizures and SUDEP in mice. Importantly, to our knowledge, this work provides the first demonstration of impaired I_Na_ density in acutely isolated fluorescent reporter‐labeled PV + interneurons in a VGSC gene‐linked DEE mouse model. Excitatory/inhibitory imbalance has been described in a variety of epilepsy models, with *Scn1a*‐linked DS mice serving as the first example of impaired inhibitory interneuron excitability driving epileptogenesis.^31^ Evidence of comparatively higher levels of *Scn1a* expression in GABAergic interneurons over pyramidal neurons, data from cell type selective *Scn1a* deletion studies, and demonstration of interneuron hypoexcitability have provided a framework for disinhibition as an underlying mechanism of DS, although age‐ and strain‐dependent increases in pyramidal neuron hyperexcitability have been reported.^15^ *SCN2A*, *SCN3A,* and *SCN8A* have comparatively higher levels of expression in pyramidal vs. GABAergic interneurons, and DEE variants in these genes have been shown to result in pyramidal neuron hyperexcitability via increased persistent, or in some cases, resurgent, I_Na_.^7^, ^26^ Here, in *Scn1b^−/−^* mice, we observed reduced I_Na_ density in PV + neurons as well as reduced transient and persistent I_Na_ in a subset of pyramidal neurons, reflecting the known roles of β1/β1B subunits in chaperoning VGSC α subunits to the plasma membrane ^3^, and presenting a novel mechanism of DEE that is distinct from either DS or EIEE13, with excitability defects impacting the excitability of both pyramidal neurons and PV + interneurons.

Our data showing that *Scn1b* deletion impairs the excitability of PV + interneurons and alters the excitability of pyramidal neurons suggest that *Scn1b*‐linked early infantile DEE pathogenesis and *Scn1a*‐linked DS and VGSC GOF pathogenesis may share common elements. Importantly, however, there remain critical differences between the models that inform current and future treatment strategies. *Scn1b^‐/‐^* mice have a more severe phenotype than *Scn1a^+/−^* or *Scn8a* GOF DEE mice,^15^, ^32^ with earlier age of seizure onset and 100% lethality by the third postnatal week. Deletion of *Mapt*, the gene encoding the microtubule‐binding protein Tau, was shown to attenuate hyperexcitability and prevent disease in the *Scn1a^R1407X/+^* mouse model of DS.^33^ We reported that intercrossing *Scn1b^+/−^* mice with *Mapt^+/−^* mice to generate *Scn1b^−/−^/Mapt^+/+^* and *Scn1b^−/−^/Mapt^−/−^* progeny had no impact on seizures or survival of *Scn1b^−/−^* mice.^34^

In other work, we reported that the time courses of maturation of neuronal GABAergic signaling in neocortical layer II/III and hippocampal CA1 or CA3 pyramidal cells are delayed in both the *Scn1b^−/−^* and *Scn1a^+/−^* mouse models of DEE, such that GABAergic signaling remains excitatory in these brain regions for significantly longer periods of development compared to WT.^28^ This important point should be considered in the context of both DS and EIEE52 when discussing the effects of disinhibition, in that defects are not limited to impaired PV + neuron firing but also that inhibitory neurotransmission is further dampened by the impaired capacity of postsynaptic cells to receive these inputs as inhibitory. We found that the mean reversal potential for GABA in *Scn1a* ^+/−^ neurons was less depolarized than that of *Scn1b* ^−/−^ neurons over a similar developmental time range, again suggesting that these two DEE models exhibit similarities with respect to impaired inhibition, but that the *Scn1b^−/−^* model is more severe. Taken together, these results show that there remain critical differences in the response to disease mitigating interventions and the pathological time courses of *Scn1a*‐ and *Scn1b*‐linked DEE, suggesting that treatment strategies developed for DS patients may not be sufficient for EIEE52 patients and alternative or adjunct treatment options should be explored. In particular, the presence of pyramidal neuron hyperexcitability at low current injections in *Scn1b^−/−^* mice suggests that treatment with K^+^ channel activators may be beneficial in EIEE52 patients, as suggested previously,^22^ whereas VGSC blockers should remain contraindicated, as is the case in DS patients.^35^

Our previous observation of neuronal pathfinding defects in *Scn1b* ^−/−^ brain prior to the onset of seizures led us to propose that altered brain development underlies epileptogenesis in EIEE52.^21^ In other work, a developmental increase in input resistance in pyramidal neurons at specific circuit points was proposed to lead to epilepsy in the related *Scn1b^CW/CW^* mouse model of DEE.^22^ More recent work from our laboratory, however, made us reconsider our previous hypothesis. We demonstrated that inducible deletion of *Scn1b* in adult mouse brain neurons using a Cre‐lox strategy, following normal development, resulted in severe epilepsy and SUDEP in 100% of mice within 3 weeks, suggesting that neither of the previously proposed mechanisms were sufficient to explain the basis of network hyperexcitability.^24^ The developmentally regulated neuronal pathfinding and fasciculation defects we reported previously in *Scn1b^−/−^* cerebellum and corticospinal tract, coupled with the region‐specific cortical pyramidal neuron hyperexcitability observed in this study may contribute to the increased severity of *SCN1B*‐linked early infantile DEE in terms of seizure onset, seizure severity, or severity of co‐morbidities, including profound motor and cognitive delays, intellectual disability, autism spectrum disorders, and hearing loss in patients. Given that both *Scn1b^−/−^* pyramidal and PV + neurons exhibit similar decreases in I_Na_, the importance of altered K^+^ currents, developmental delay, altered GABA reversal potential, cellular context, and other effects may differentiate the signaling mechanisms leading to impaired firing of PV + neurons and the subsequent impact on network function. Further work on this mouse model may reveal crucial insights into the diverse mechanisms that coordinate network excitability. Finally, our results suggest that the choice of pharmacological treatment regimens for EIEE52 should be re‐interpreted in light of the involvement of both excitatory and inhibitory neuronal deficits, which reflect divergent defects from each of the other major classes of VGSC related DEEs.

## Author Contributions

JMH performed electrophysiology and analysis for all figures and wrote the manuscript. HAO performed all histochemistry and analysis. CC and KA performed mouse breeding, genotyping, and record keeping. JMH, CC, and KA monitored mouse survival. YY contributed electrophysiology for Fig. 2. AB and ND aided in sample preparation. LFL‐S served as a co‐mentor with LLI to JMH. LLI wrote the manuscript and was responsible for overseeing all experiments.

## Conflicts of Interest

None.

## Supporting information

Movie S1. Example of a spontaneous convulsive seizure in a P17 *Scn1b^Fl/Fl^*/PV‐Cre mouse.
